# Arrow Contraction and Expansion in Tropical Diagrams

**DOI:** 10.3390/e25121637

**Published:** 2023-12-08

**Authors:** Rostislav Matveev, Jacobus W. Portegies

**Affiliations:** 1 Max Planck Institute for Mathematics in the Sciences, 04103 Leipzig, Germany; 2 Department of Mathematics and Computer Science, Eindhoven University of Technology, 5612 AZ Eindhoven, The Netherlands

**Keywords:** tropical probability, entropic cone

## Abstract

Arrow contraction applied to a tropical diagram of probability spaces is a modification of the diagram, replacing one of the morphisms with an isomorphism while preserving other parts of the diagram. It is related to the rate regions introduced by Ahlswede and Körner. In a companion article, we use arrow contraction to derive information about the shape of the entropic cone. Arrow expansion is the inverse operation to the arrow contraction.

## 1. Introduction

In [1], we have initiated the theory of *tropical probability spaces* for the systematic study of information optimization problems in information theory and artificial intelligence, such as those arising in robotics [2], neuroscience [3], artificial intelligence [4], variational autoencoders [5], information decomposition [6], and causal inference [7]. In [8], we applied the techniques to derive a dimension-reduction result for the entropic cone of four random variables.

Two of the main tools used for the latter are what we call *arrow contraction* and *arrow expansion*. They are formulated for tropical commutative diagrams of probability spaces. Tropical diagrams are points in the asymptotic cone of the metric space of commutative diagrams of probability spaces endowed with the asymptotic entropy distance. Arrows in diagrams of probability spaces are (equivalence classes of) measure-preserving maps.

Arrow contraction and expansion take a commutative diagram of probability spaces as input, modify it, but preserve important properties of the diagram. The precise results are formulated as Theorems 3 and 4 in the main text. Their formulation requires language, notation, and definitions that we review in Section 2.

However, to give an idea of the results in this paper, we now present two examples. For basic terminology and notations used in these examples below, the reader unfamiliar with them is referred either to Section 2 of the present article or in the introductory material in the article [9].

### 1.1. Two Examples

#### 1.1.1. Arrow Contraction and Expansion in a Two-Fan

Suppose we are given a fan $Z=(X\overset{}{\leftarrow }Z\overset{}{\to }Y)$, and we would like to complete it to a diamond
(1)
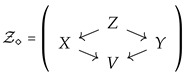

such that the entropy of *V*, denoted by [V], equals the mutual information [X:Y] between *X* and *Y*, i.e., we would like to realize the mutual information between *X* and *Y* by a pair of reductions $X\overset{}{\to }V$ and $Y\overset{}{\to }V$. This is not always possible, not even approximately. The Gacs-Körner Theorem [10] describes when such exact realization of mutual information is possible.

Arrow contraction instead produces another fan $Z^{\prime }=\left(X\overset{}{\leftarrow }Z^{\prime }\overset{}{\to }V\right)$, such that the reduction $Z^{\prime }\overset{}{\to }X$ is an isomorphism and the relative entropy [X|V] of *X* given *V* equals [X|Y]. By collapsing this reduction, we obtain as a diagram just the reduction $X\overset{}{\to }V$. If necessary, we can keep the original spaces *Z* and *Y* in the modified diagram obtaining the “broken diamond” diagram

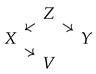

such that [V]=[X:Y]. Of course, no special technique is necessary to achieve this result since it is easy to find a reduction from a tropical space [X] to another tropical probability space with the prespecified entropy, as long as the Shannon inequalities are not violated.

However, a similar operation becomes non-trivial and in fact impossible without passing to the tropical limit, if instead of a single space *X*, there is a more complex sub-diagram as in the example in the next subsection.

To explain how arrow expansion works, we start with the chain of reductions $Z\overset{}{\to }X\overset{}{\to }V$. Can we extend it to a diamond, as in (1), so that [X:Y|V]=0? This is again not possible, in general. However, if we pass to tropical diagrams, then such an extension always exists.

#### 1.1.2. One More Example of Arrow Expansion and Contraction

**Figure 1:**
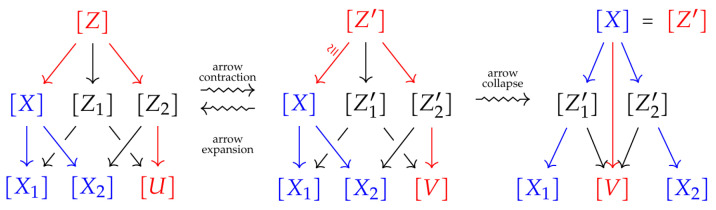
Arrow contraction and expansion in a $\Lambda_{3}$-diagram. The fan $\left(\left[X\right]\overset{}{\leftarrow }\left[Z\right]\overset{}{\to }\left[U\right]\right)$ (shown in red in the Figure) is admissible. Spaces $\left[Z_{1}\right]$, $\left[Z_{2}\right]$ and [Z] belong to the co-ideal $\left\lfloor U\right\rfloor$. After the operation the part of the diagram shown in blue in the Figure is left unmodified.

Consider a diagram presented in Figure 1. Such a diagram is called a $\Lambda_{3}$-diagram. We would like to find a reduction $X\overset{}{\to }V$ so that [X|U]=[X|V]. It is not possible to achieve this within the realm of diagrams of classical probability spaces. But once we pass to the tropical limit, the reduction $\left[X\right]\overset{}{\to }\left[V\right]$ can be found by contracting and then collapsing the arrow $\left[Z\right]\overset{}{\to }\left[X\right]$, as shown in Figure 1.

Arrow contraction is closely related to the Shannon channel coding theorem. This is perhaps most obvious from the proof. Furthermore, arrow contraction has connections with rate regions, as introduced by Ahlswede and Körner, see [11,12]. These results by Ahlswede and Körner were applied by [13], resulting in a new non-Shannon information inequality. Moreover, in [13], a new proof was given of the results; this new proof is similar to the proof of the arrow contraction result in the present paper.

The main contribution of our work lies in the fact that we prove a much stronger preservation of properties of the diagram under arrow contraction.

## 2. Preliminaries

### 2.1. Probability Spaces and Their Diagrams

Our main objects of study will be *commutative diagrams of probability spaces*. A *finite probability space X* is a set with a probability measure on it, supported on a finite set. We denote by |X| the cardinality of the support of the measure. The statement x∈X means that point *x* is an atom with positive weight in *X*. For details see [1,9,14].

**Figure 2:**
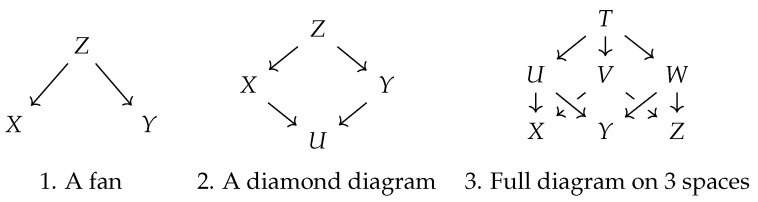
Examples of diagrams of probability spaces.

Examples of commutative diagrams of probability spaces are shown in Figure 2. The objects in such diagrams are finite probability spaces and morphisms are equivalence classes of measure-preserving maps. Two such maps are considered to be equivalent if they coincide on a set of full measurements. To record the combinatorial structure of a commutative diagram, i.e., the arrangement of spaces and morphisms, we use *indexing categories*, which are finite poset categories satisfying an additional property, which we describe below.

#### 2.1.1. Indexing Categories

A *poset category* is a finite category such that there is at most one morphism between any two objects either way.

For a pair of objects k,l in a poset category $G=\left\{i;\;\gamma_{ij}\right\}$, such that there is a morphism $\gamma_{kl}$ in G, we call *k* an ancestor of *l* and *l* a descendant of *k*. The set of all ancestors of an object *k* together with all the morphisms between them is itself a poset category and will be called a *co-ideal* generated by *k* and denoted by $\left\lfloor k\right\rfloor$. Co-ideals are also sometimes called *filters*. Similarly, a poset category consisting of all descendants of k∈G and morphisms between them will be called an *ideal* generated by *k* and denoted $\left\lceil k\right\rceil$.

An *indexing category* $G=\left\{i;\;\gamma_{ij}\right\}$ used for indexing diagrams is a poset category satisfying the following additional property: for any pair of objects $i_{1},i_{2}\in G$ the the intersection of co-ideals is also a co-ideal generated by some object $i_{3}\in G$,

$$\left\lfloor i_{1}\right\rfloor \cap \left\lfloor i_{2}\right\rfloor =\left\lfloor i_{3}\right\rfloor$$

In other words, for any pair of objects $i_{1},i_{2}\in G$ there exists a *least common ancestor* $i_{3}$, i.e., $i_{3}$ is an ancestor to both $i_{1}$ and $i_{2}$ and any other common ancestor is also an ancestor of $i_{3}$. Any indexing category is *initial*, i.e., there is a (necessarily unique) *initial* object $\hat{ı}$ in it, which is the ancestor of any other object in G, in other words $G=\left\lceil \hat{ı}\right\rceil$.

A *fan* in a category is a pair of morphisms with the same domain. Such a diagram is also called a *span* in some literature on Category Theory. A fan $\left(i\overset{}{\leftarrow }k\overset{}{\to }j\right)$ is called *minimal*, if for any other fan $\left(i\overset{}{\leftarrow }l\overset{}{\to }j\right)$ included in a commutative diagram

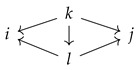

the vertical morphism $\left(k\overset{}{\to }l\right)$ must be an isomorphism. Any indexing category also satisfies the property that, for any pair of objects in it, there exists a unique minimal fan with target objects of the given ones.

This terminology will also be applied to diagrams of probability spaces indexed by G. Thus, given a space *X* in a G-diagram, we can talk about its ancestors, descendants, co-ideal $\left\lfloor X\right\rfloor$, and ideal $\left\lceil X\right\rceil$. We use square brackets to denote tropical diagrams and spaces in them. For the (co-)ideals in tropical diagrams, in order to unclutter notations, we will write

$$\left\lfloor X\right\rfloor :=\left\lfloor [X]\right\rfloor \;\mathrm{and}\;\left\lceil X\right\rceil :=\left\lceil [X]\right\rceil$$

#### 2.1.2. Diagrams

For an indexing category $G=\left\{i;\;\gamma_{ij}\right\}$ and a category Cat, a commutative G-diagram $X=\left\{X_{i};\;\chi_{ij}\right\}$ is a functor $X:G\overset{}{\to }\mathrm{Cat}$. A diagram X is called *minimal* if it maps minimal fans in G to minimal fans in Cat.

A *constant* G*-diagram* denoted $X^{G}$ is a diagram where all the objects equal to *X*, and all morphisms are identities.

Important examples of indexing categories are a two-fan, a diamond category, a full category $\Lambda_{n}$ on *n* spaces, chains $C_{n}$. For detailed descriptions and more examples, the reader is referred to the articles cited at the beginning of this section.

### 2.2. Tropical Diagrams

#### 2.2.1. Intrinsic Entropy Distance

For a fixed indexing category G, the space of commutative G-diagrams will be denoted by $\mathrm{Prob}\langle G\rangle$. Evaluating entropy on every space in a G diagram gives a map

$${\mathrm{Ent}}_{*}:\mathrm{Prob}\langle G\rangle \overset{}{\to }R^{G}$$

where the target space $R^{G}$ is the space of real-valued functions on objects of G. We endow this space with the $\ell^{1}$-norm. For a fan $F=(X\overset{}{\leftarrow }Z\overset{}{\to }Y)$ of G-diagrams we define the entropy distance between its terminal objects by

$$\mathrm{kd}\left(F\right):={∥{\mathrm{Ent}}_{*}Z-{\mathrm{Ent}}_{*}X∥}_{1}+{∥{\mathrm{Ent}}_{*}Z-{\mathrm{Ent}}_{*}Y∥}_{1}$$

and the intrinsic entropy distance between two arbitrary G-diagrams by

$$k\left(X,Y\right):=\inf\left\{\mathrm{kd}\left(F\right)\;:\;F=\left(X\overset{}{\leftarrow }Z\overset{}{\to }Y\right)\right\}$$

This intrinsic version of the entropy distance was introduced in [15,16]. The triangle inequality for k and various other properties are discussed in [1].

In the same article, a useful estimate for the intrinsic entropy distance called the Slicing Lemma is also proven. The following corollary ([1], Corollary 3.10(1)) of the Slicing Lemma will be used in the next section.

**Proposition 1.**  *Let G be an indexing category, $X,Y\in \mathrm{Prob}\langle G\rangle$ and U∈Prob included in a pair of two fans*


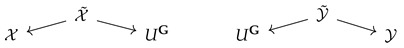


*Then*

$$k\left(X,Y\right)\leq \int_{U}k\left(X|u,Y|u\right)\;dp_{U}\left(u\right)+2\cdot \left[\;\left[G\right]\;\right]\cdot \mathrm{Ent}\left(U\right)$$

#### 2.2.2. Tropical Diagrams

Points in the asymptotic cone of $\left(\mathrm{Prob}\langle G\rangle ,k\right)$ are called tropical G-diagrams and the space of all tropical G-diagrams, denoted Prob[G], is endowed with the *asymptotic entropy distance*. We explain this now in more detail, and a more extensive description can be found in [14].

To describe points in Prob[G] we consider *quasi-linear* sequences $\bar{X}:=\left(X\left(n\right):\;n\in N\right)$ of G-diagrams. That is, we fix a “slowly growing” increasing function $\varphi :R_{\geq 0}\overset{}{\to }R$ satisfying

$$t\cdot \int_{t}^{\infty }\frac{\varphi (t)}{t^{2}}\;dt\leq D_{\varphi }\cdot \varphi \left(t\right)$$

for some constant $D_{\varphi }>0$ and any t>1. We call a sequence $\bar{X}:=\left(X\left(n\right):\;n\in N\right)$ φ-quasi-linear if it satisfies the bound for all m,n∈N

$$\kappa \left(X(n+m),X(n)⊗X(m)\right)\leq C\cdot \varphi \left(n+m\right)$$

We have shown in [14] that the space Prob[G] does not depend on the choice of function φ as long as it is not zero. The space of all such sequences is endowed with the *asymptotic entropy distance* defined by

$$\kappa \left(\bar{X},\bar{Y}\right):=\lim_{n\overset{}{\to }\infty }\frac{1}{n}k\left(X(n),Y(n)\right)$$

A tropical diagram [X] is defined to be an equivalence class of such sequences, where two sequences $\bar{X}$ and $\bar{Y}$ are equivalent if $\kappa (\bar{X},\bar{Y})=0$. The space Prob[G] carries the asymptotic entropy distance and has the structure of a $R_{\geq 0}$-semi-module—one can take linear combinations with non-negative coefficients of tropical diagrams. The linear entropy functional ${\mathrm{Ent}}_{*}:\mathrm{Prob}\left[G\right]\overset{}{\to }R^{G}$ is defined by

$${\mathrm{Ent}}_{*}\left[X\right]:=\lim_{n\overset{}{\to }\infty }\frac{1}{n}{\mathrm{Ent}}_{*}X\left(n\right)$$

A detailed discussion about tropical diagrams can be found in [14]. In the cited article, we show that the space Prob[G] is metrically complete and isometrically isomorphic to a closed convex cone in some Banach space.

For $G=C_{k}$ a *chain category*, containing *k* objects $\left\{1,\ldots ,k\right\}$ and unique morphism $i\overset{}{\to }j$ for every pair i≥j, we have shown in [14] that the space $\mathrm{Prob}[C_{k}]$ is isomorphic to the following cone in $\left(R^{k},{∥\;\cdot \;∥}_{1}\right)$

$$\mathrm{Prob}\left[C_{k}\right]≅\left\{\left(\begin{array}{c} \;\;x_{1}\;\;\\ \vdots \\ \;\;x_{k}\;\; \end{array}\right)\;:\;0\leq x_{1}\leq \cdots \leq x_{k}\right\}$$

The isomorphism is given by the entropy function. Thus, we can identify tropical probability spaces (elements in $\mathrm{Prob}[C_{1}]$) with non-negative numbers via entropy. We will simply write [X] to mean the entropy of the space [X]. Along these lines, we also adopt the notations [X|Y], [X:Y] and [X:Y|Z] for the conditional entropy and mutual information for the tropical spaces included in some diagrams.

### 2.3. Asymptotic Equipartition Property for Diagrams

#### 2.3.1. Homogeneous Diagrams

A G-diagram X is called *homogeneous* if the automorphism group Aut(X) acts transitively on every space in X. Homogeneous probability spaces are uniform. For more complex indexing categories, this simple description is not sufficient.

#### 2.3.2. Tropical Homogeneous Diagrams

The subcategory of all homogeneous G-diagrams will be denoted $\mathrm{Prob}{\langle G\rangle }_{h}$ and we write $\mathrm{Prob}{\langle G\rangle }_{h,m}$ for the category of minimal homogeneous G-diagrams. These spaces are invariant under the tensor product. Thus, they are metric Abelian monoids.

Passing to the tropical limit, we obtain spaces of tropical (minimal) homogeneous diagrams that we denote $\mathrm{Prob}{\left[G\right]}_{h}$ and $\mathrm{Prob}{\left[G\right]}_{h,m}$.

#### 2.3.3. Asymptotic Equipartition Property

In [1] the following theorem is proven

**Theorem 1.**  *Suppose $X\in \mathrm{Prob}\langle G\rangle$ is a G-diagram of probability spaces for some fixed indexing category G. Then, there exists a sequence $\bar{H}={\left(H_{n}\right)}_{n=0}^{\infty }$ of homogeneous G-diagrams such that*

(2) $$\frac{1}{n}k\left(X^{n},H_{n}\right)\leq C(|X_{0}\left|,\left[\;\left[G\right]\;\right]\right)\cdot \sqrt{\frac{\ln^{3}n}{n}}$$

*where $C(|X_{0}|,\left[\;\left[G\right]\;\right])$ is a constant only depending on $|X_{0}|$ and [[G]].*

The approximating sequence of homogeneous diagrams is evidently quasi-linear with the defect bounded by the admissible function

$$\varphi \left(t\right):=2C(|X_{0}\left|,\left[\;\left[G\right]\;\right]\right)\cdot t^{3/4}\geq 2C(|X_{0}\left|,\left[\;\left[G\right]\;\right]\right)\cdot t^{1/2}\cdot \ln^{3/2}t$$

Thus, Theorem 1 above states that $L\left(\mathrm{Prob}\langle G\rangle \right)\subset \mathrm{Prob}{\left[G\right]}_{h}$. On the other hand, we have shown in [14] that the space of linear sequences $L(\mathrm{Prob}\langle G\rangle )$ is dense in Prob[G]. Combining the two statements, we obtain the following theorem.

**Theorem 2.**  *For any indexing category G, the space $\mathrm{Prob}{\left[G\right]}_{h}$ is dense in Prob[G]. Similarly, the space $\mathrm{Prob}{\left[G\right]}_{h,m}$ is dense in $\mathrm{Prob}{\left[G\right]}_{m}$.*

It is possible that the spaces $\mathrm{Prob}{\left[G\right]}_{h}$ and Prob[G] coincide. At this time, we have neither a proof nor a counterexample to this conjecture.

### 2.4. Conditioning in Tropical Diagrams

For a tropical G-diagram [X] containing a space [U] we defined a conditioned diagram [X|U]. It can be understood as the tropical limit of the sequence $\left(X\left(n\right)|u_{n}\right)$, where (X(n)) is the homogeneous approximation of [X], U(n) is the space in X(n) that corresponds to [U] under combinatorial isomorphism and $u_{n}$ is any atom in U(n).

We have shown in [9] that operation of conditioning is Lipschitz-continuous with respect to the asymptotic entropy distance.

## 3. Arrow Contraction

### 3.1. Arrow Collapse, Arrow Contraction, and Arrow Expansion

#### 3.1.1. Prime Morphisms

A morphism $\gamma_{ij}:i\overset{}{\to }j$ in an indexing category $G=\left\{i;\gamma_{ij}\right\}$ will be called *prime* if it cannot be factored into a composition of two non-identity morphisms in G. A morphism in a G-diagram indexed by a prime morphism in G will also be called *prime*.

#### 3.1.2. Arrow Collapse

Suppose Z is a G-diagram such that for some pair i,j∈G, the prime morphism $\zeta_{ij}:Z_{i}\overset{}{\to }Z_{j}$ is an isomorphism. *Arrow collapse* applied to Z results in a new diagram $Z^{\prime }$ obtained from Z by identifying $Z_{i}$ and $Z_{j}$ via the isomorphism $\zeta_{ij}$. The combinatorial type of $Z^{\prime }$ is different from that of Z. The spaces $Z_{i}$ and $Z_{j}$ are replaced by a single space, and the new space will inherit all the morphisms in Z with targets and domains $Z_{i}$ and $Z_{j}$.

#### 3.1.3. Arrow Contraction and Expansion

*Arrow contraction and expansion* are two operations on tropical G-diagrams. Roughly speaking, arrow contraction applied to a tropical G-diagram [Z] results in another tropical G-diagram $\left[Z^{\prime }\right]$ such that one of the arrows becomes an isomorphism, while some parts of the diagram are not modified. Arrow expansion is an inverse operation to arrow contraction.

#### 3.1.4. Admissible and Reduced Sub-Fans

An *admissible fan* in a G-diagram Z is a minimal fan $X\overset{}{\leftarrow }Z\overset{}{\to }U$, such that *Z* is the initial space of Z and any space in Z belongs either to the co-ideal $\left\lceil X\right\rceil$ or ideal $\left\lfloor U\right\rfloor$. For example, in the left-most diagram of Figure 1, the fan $X\overset{}{\leftarrow }Z\overset{}{\to }U$ is admissible, while $X_{1}\overset{}{\leftarrow }Z_{1}\overset{}{\to }U$ or $X\overset{}{\leftarrow }Z\overset{}{\to }Z_{2}$ are not.

An admissible fan $X\overset{}{\leftarrow }Z\overset{}{\to }U$ in a diagram will be called *reduced* if the morphism $Z\overset{}{\to }X$ is an isomorphism.

### 3.2. The Contraction Theorem

Our aim is to prove the following theorem.

**Theorem 3.**  *Let $\left(\left[X\right]\overset{}{\leftarrow }\left[Z\right]\overset{}{\to }\left[U\right]\right)$ be an admissible fan in some tropical G-diagram [Z]. Then for every ε>0 there exists a G-diagram $\left[Z^{\prime }\right]$ containing an admissible fan $\left(\left[X^{\prime }\right]\overset{}{\leftarrow }\left[Z^{\prime }\right]\overset{}{\to }\left[U^{\prime }\right]\right)$, corresponding to the original admissible fan through the combinatorial isomorphism, such that, with the notations $X=\left\lceil X\right\rceil$ and $X^{\prime }=\left\lceil X^{\prime }\right\rceil$, the diagram $\left[Z^{\prime }\right]$ satisfies*

*(i)* 
$\kappa (\left[X^{\prime }|U^{\prime }\right],\left[X|U\right])\leq \varepsilon$

*(ii)* 
$\kappa (X^{\prime },X)\leq \varepsilon$

*(iii)* 
$[Z^{\prime }|X^{\prime }]\leq \varepsilon$

It is not clear that constructing diagrams $Z^{\prime }$ as in the theorem above for a sequence of values of parameter ε decreasing to 0, we can obtain a convergent sequence in Prob[G] with the limiting diagram satisfying conclusions of the theorem with ε=0. If Prob[G] were a locally compact space, which is an open question at the moment. The convergence would be guaranteed, and then ε in the theorem above could be replaced by 0.

The proof of Theorem 3 is based on the following proposition, which will be proven in Section 5.

**Proposition 2.**  *Let $\left(X_{0}\overset{}{\leftarrow }Z_{0}\overset{}{\to }U\right)$ be an admissible fan in some* homogeneous *G-diagram of probability spaces Z. Then there exists a G-diagram $Z^{\prime }$ containing the admissible fan $\left(X_{0}^{\prime }\overset{}{\leftarrow }Z_{0}^{\prime }\overset{}{\to }U^{\prime }\right)$ such that, with the notations $X:=\left\lceil X_{0}\right\rceil$ and $X^{\prime }:=\left\lceil X_{0}^{\prime }\right\rceil$, it holds that*

*(1)*  *$X|u=X^{\prime }|u^{\prime }$ for any u∈U and $u^{\prime }\in U^{\prime }$.*
*(2)*  $\kappa \left(X,X^{\prime }\right)\leq k\left(X,X^{\prime }\right)\leq 20\cdot \left[\;\left[G\right]\;\right]$
*(3)*  $\left[Z_{0}^{\prime }|X_{0}^{\prime }\right]\leq 4\ln\ln\left|X_{0}\right|$

**Proof of Theorem 3.**  First, we assume that [Z] is a homogeneous tropical diagram. It means that it can be represented by a quasi-linear sequence ${\left(Z\left(n\right)\right)}_{n\in N_{0}}$ of homogeneous diagrams, with defect of the sequence bounded by the function $\varphi \left(t\right):=C\cdot t^{3/4}$ for some C≥0. This means that for any m,n∈N

$$\begin{array}{cc} \kappa (Z(m)⊗Z(n),Z(m+n)) & \leq \varphi (m+n)\\ \kappa (Z^{m}\left(n\right),Z\left(m\cdot n\right)) & \leq D_{\varphi }\cdot m\cdot \varphi \left(n\right) \end{array}$$

where $D_{\varphi }$ is some constant depending on φ, see [14].

Fix a number n∈N and apply Proposition 2 to the *homogeneous* diagram Z(n), containing the admissible fan $X_{0}\left(n\right)\overset{}{\leftarrow }Z_{0}\left(n\right)\overset{}{\to }U\left(n\right)$ and sub-diagram $X\left(n\right)=\left\lceil X_{0}\left(n\right)\right\rceil$. As a result, we obtain a diagram $Z^{\prime \prime }$ containing the fan $X_{0}^{\prime \prime }\overset{}{\leftarrow }Z_{0}^{\prime \prime }\overset{}{\to }U^{\prime \prime }$ and the sub-diagram $X^{\prime \prime }=\left\lceil X_{0}^{\prime \prime }\right\rceil$, such that

(3) $$\begin{array}{cc} X^{\prime \prime }\left|u^{\prime \prime }=X\left(n\right)\right|u\; & \;\mathrm{for}\;\mathrm{any}\;u^{\prime \prime }\in U^{\prime \prime }\;\mathrm{and}\;u\in U(n)\\ \kappa \left(X^{\prime \prime },X\left(n\right)\right)\leq 20\left[\;\left[G\right]\;\right] & \;\\ \left[Z_{0}^{\prime \prime }|X_{0}^{\prime \prime }\right]\leq 4\ln\ln\left|X_{0}\left(n\right)\right| & \; \end{array}$$

Recall that for a diagram A of probability spaces, we denote by $\vec{A}$ the tropical diagram represented by the linear sequence $\left(A^{k}\;:\;k\in N_{0}\right)$. As an element of a closed convex cone Prob[G], it can be scaled by an arbitrary non-negative real number; see, for instance, Section 2.3.5 in [14]. For example, $\frac{1}{n}\vec{A}$ is represented by the sequence $\left(A^{\left\lfloor \frac{k}{n}\right\rfloor }\;:\;k\in N_{0}\right)$.

Define the two tropical diagrams

$$\begin{array}{cc} \left[Z^{\prime }\right] & :=\frac{1}{n}\vec{Z^{\prime \prime }}\\ {}[\tilde{Z}] & :=\frac{1}{n}\vec{Z(n)} \end{array}$$

Since $X^{\prime \prime }|u^{\prime \prime }$ does not depend on $u^{\prime \prime }$ and X(n)|u does not depend on *u* we have $\left[X^{\prime }|U^{\prime }\right]=\left(1/n\right)\cdot \vec{\left(X^{\prime \prime }|u^{\prime \prime }\right)}$ and $\left[\tilde{X}|\tilde{U}\right]=\left(1/n\right)\cdot \vec{\left(X(n)|u\right)}$. From (3), we obtain

(4) $$\begin{array}{cc} \; & \left[X^{\prime }|U^{\prime }\right]=\left[\tilde{X}|\tilde{U}\right]\\ \; & \kappa \left(\left[X^{\prime }\right],\left[\tilde{X}\right]\right)\leq \frac{20[\;[G]\;]}{n}\\ \; & \left[Z_{0}^{\prime }|X_{0}^{\prime }\right]\leq \frac{4\ln\ln|X_{0}\left(n\right)|}{n} \end{array}$$

The distance between $\left[\tilde{Z}\right]$ and [Z] can be bounded as follows

(5) $$\begin{array}{cc} \kappa \left(\left[\tilde{Z}\right],\left[Z\right]\right)\; & =\frac{1}{n}\kappa \left(\vec{Z(n)},n\cdot \left[Z\right]\right)=\frac{1}{n}\lim_{m\overset{}{\to }\infty }\frac{1}{m}\kappa \left(Z^{m}\left(n\right),Z\left(m\cdot n\right)\right)\\ \; & \leq \frac{1}{n}D_{\varphi }\cdot \varphi \left(n\right) \end{array}$$

This also implies

(6) $$\kappa \left(\left[\tilde{X}\right],\left[X\right]\right)\leq \frac{1}{n}D_{\varphi }\cdot \varphi \left(n\right)$$

Since conditioning is a Lipschitz-continuous operation with Lipschitz constant 2, we also have

(7) $$\kappa \left(\left[\tilde{X}|\tilde{U}\right],\left[X|U\right]\right)\leq \frac{2}{n}D_{\varphi }\cdot \varphi \left(n\right)$$

Combining the estimates in (4)–(7) we obtain

$$\begin{array}{cc} \; & \kappa \left(\left[X^{\prime }|U^{\prime }\right],\left[X|U\right]\right)\leq 2D_{\varphi }\cdot \frac{\varphi (n)}{n}\\ \; & \kappa \left(\left[X^{\prime }\right],\left[X\right]\right)\leq \frac{20[\;[G]\;]}{n}+D_{\varphi }\frac{\varphi (n)}{n}\\ \; & \left[Z_{0}^{\prime }|X_{0}^{\prime }\right]\leq \frac{4\ln\ln|X_{0}\left(n\right)|}{n}+2D_{\varphi }\frac{\varphi (n)}{n} \end{array}$$

Please note that $|X_{0}\left(n\right)|$ grows at most exponentially (it is bounded by $e^{n(\left[X_{0}\right]+C)}$ for some *C*) and φ is a strictly sub-linear function. Thus, by choosing sufficiently large *n* depending on the given ε>0, we obtain $\left[Z^{\prime }\right]$, satisfying conclusions of the theorem for homogeneous [Z].

To prove the theorem in full generality, observe that all the quantities on the right-hand side of the inequalities are Lipschitz-continuous. Since $\mathrm{Prob}{\left[G\right]}_{h}$ is dense in Prob[G] the theorem extends to any [Z] by first approximating it with any precision by a homogeneous configuration and applying the argument above. □

### 3.3. The Expansion Theorem

The following theorem is complementary to Theorem 3. The expansion applied to a diagram containing a reduced admissible fan produces a diagram with an admissible fan, such that the contraction of it is the original diagram. Thus, arrow expansion is a right inverse of the arrow contraction operation.

In general, contraction erases some information stored in the diagram, so there are many right inverses. We prove the theorem below by providing a simple construction of one such right inverse.

**Theorem 4.**  *Let $\left(\left[X\right]\overset{}{\leftarrow }\left[Z^{\prime }\right]\overset{}{\to }\left[U^{\prime }\right]\right)$ be a reduced admissible fan in some tropical G-diagram $\left[Z^{\prime }\right]$ and λ>0. Let $\left[X\right]:=\left\lceil X\right\rceil$. Then there exists a G-diagram [Z] containing the copy of [X], such that the corresponding admissible fan $\left(\left[X\right]\overset{}{\leftarrow }\left[Z\right]\overset{}{\to }\left[U\right]\right)$ has [Z|X]=λ and $\left[X|U\right]=\left[X|U^{\prime }\right]$.*

**Proof.**  Let [W] be a tropical probability space with entropy equal to λ. For any reduction of tropical spaces $\left[A\right]\overset{}{\to }\left[B\right]$, there are natural reductions

$$\begin{array}{cc} \left([A]+[W]\right) & \overset{}{\to }\left(\left[B\right]+\left[W\right]\right)\\ \left([A]+[W]\right) & \overset{}{\to }\left[W\right] \end{array}$$

We construct the diagram [Z] by replacing every space [V] in the ideal $\left\lfloor U^{\prime }\right\rfloor$ with [U]+[W]. Every morphism $\left[V_{1}\right]\overset{}{\to }\left[V_{2}\right]$ within $\left\lfloor U^{\prime }\right\rfloor$ is replaced by

$$\left(\left[V_{1}\right]+\left[W\right]\right)\overset{}{\to }(\left[V_{2}\right]+\left[W\right])$$

And any morphism from [V] in $\left\lfloor U^{\prime }\right\rfloor$ to a space [Y] in $\left\lceil X\right\rceil$ is replaced by a composition

$$\left([V]+[W]\right)\overset{}{\to }\left[V\right]\overset{}{\to }\left[Y\right]$$

Clearly, the resulting diagram satisfies the conclusion of the theorem. □

The rest of the article is devoted to the development of the necessary tools and the proof of Proposition 2.

## 4. Local Estimate

In this section, we derive a bound, very similar to Fano’s inequality, on the intrinsic entropic distance between two diagrams of probability spaces with the same underlying diagram of sets. The bound will be in terms of the total variation distance between two distributions corresponding to the diagrams of probability spaces. It will be used in the next section to prove Proposition 2.

### 4.1. Distributions

#### 4.1.1. Distributions on Sets

For a finite set *S* we denote by ΔS the collection of all probability distributions on *S* and by $∥\pi_{1}-\pi_{2}{∥}_{1}$ we denote the total variation distance between $\pi_{1},\pi_{2}\in \Delta S$.

#### 4.1.2. Distributions on Diagrams of Sets

Let Set denote the category of finite sets and surjective maps. For an indexing category G, we denote by $\mathrm{Set}\langle G\rangle$ the category of G-diagrams in Set. That is, objects in $\mathrm{Set}\langle G\rangle$ are commutative diagrams of sets indexed by the category G, the spaces in such a diagram are finite sets, and arrows represent surjective maps, subject to commutativity relations.

For a diagram of sets $S=\left\{S_{i};\sigma_{ij}\right\}$ we define the *space of distributions on the diagram* S by

$$\Delta S:=\left\{\left(\pi_{i}\right)\in \prod_{i}\Delta S_{i}\;:\;{\left(\sigma_{ij}\right)}_{*}\pi_{i}=\pi_{j}\right\}$$

where $f_{*}:\Delta S\overset{}{\to }\Delta S^{\prime }$ is the affine map induced by a surjective map $f:S\overset{}{\to }S^{\prime }$. If $S_{0}$ is the initial space of S, then there is an isomorphism

(8) $$\begin{array}{cc} \Delta S_{0} & \overset{≅}{↔}\Delta S\\ \Delta S_{0}∋\pi_{0}\; & \mapsto \left\{{\left(\sigma_{0i}\right)}_{*}\pi_{0}\right\}\in \Delta S\\ \Delta S_{0}∋\pi_{0}\; & ↤\left\{\pi_{i}\right\}\in \Delta \end{array}$$

Using the isomorphism (8) we define total variation distance between two distributions $\pi ,\pi^{\prime }\in \Delta S$ as

$${∥\pi -\pi^{\prime }∥}_{1}:={∥\pi_{0}-\pi_{0}^{\prime }∥}_{1}$$

Given a G-diagram of sets $S=\left\{S_{i};\sigma_{ij}\right\}$ and an element π∈ΔS we can construct a G-diagram of probability spaces $\left(S,\pi \right):=\left\{\left(S_{i},\pi_{i}\right);\sigma_{ij}\right\}$.

Below, we give the estimate of the entropy distance between two G-diagrams of probability spaces (S,π) and $\left(S,\pi^{\prime }\right)$ in terms of the total variation distance $∥\pi -\pi^{\prime }∥$ between distributions.

### 4.2. The Estimate

The upper bound on the entropy distance, which we derive below, has two summands. One is linear in the total variation distance with the slope proportional to the log-cardinality of $S_{0}$. The second one is super-linear in the total variation distance, but it does not depend on S. So, we have the following interesting observation: of course, the super-linear summand always dominates the linear one locally. However, as the cardinality of S becomes large, it is the linear summand that starts playing the main role. This will be the case when we apply the bound in the next section.

For α∈[0,1] consider a binary probability space with the weight of one of the atoms equal to α

$$B_{\alpha }:=\left(\left\{□,■\right\};\;p\left(□\right)=1-\alpha ,\;p\left(■\right)=\alpha \right)$$

**Proposition 3.**  *For an indexing category G, consider a G-diagram of sets $S=\left\{S_{i},\sigma_{ij}\right\}\in \mathrm{Set}\langle G\rangle$. Let $\pi ,\pi^{\prime }\in \Delta S$ be two probability distributions on S. Denote X:=(S,π), $Y:=(S,\pi^{\prime })$ and $\alpha :=\frac{1}{2}{∥\pi -\pi^{\prime }∥}_{1}$. Then*

$$k\left(X,Y\right)\leq 2\left[\;\left[G\right]\;\right]\left(\alpha \cdot \ln|S_{0}|+\mathrm{Ent}\left(B_{\alpha }\right)\right)$$

**Proof.**  To prove the local estimate, we decompose both π and $\pi^{\prime }$ into a convex combination of a common part $\hat{\pi }$ and rests $\pi^{+}$ and $\pi^{\prime +}$. The coupling between the common parts gives no contribution to the distance and the worst possible estimate on the other parts is still enough to obtain the bound in the lemma, using Proposition 1.

Let $S_{0}$ be the initial set in the diagram S. We will need the following obvious rough estimate of the entropy distance that holds for any $\pi ,\pi^{\prime }\in \Delta S$:

(9) $$k\left(X,Y\right)\leq 2\left[\;\left[G\right]\;\right]\cdot \ln|S_{0}|$$

It can be obtained by taking a tensor product for the coupling between X and Y.

Our goal now is to write π and $\pi^{\prime }$ as the convex combination of three other distributions $\hat{\pi }$, $\pi^{+}$ and $\pi^{\prime +}$ as in

$$\begin{array}{cc} \pi & =\left(1-\alpha \right)\cdot \hat{\pi }+\alpha \cdot \pi^{+}\\ \pi^{\prime } & =\left(1-\alpha \right)\cdot \hat{\pi }+\alpha \cdot \pi^{\prime +} \end{array}$$

with the smallest possible α∈[0,1].

We could do it the following way. Let $\pi_{0}$ and $\pi_{0}^{\prime }$ be the distributions on $S_{0}$ that correspond to π and $\pi^{\prime }$ under isomorphisms (8). Let $\alpha :=\frac{1}{2}{∥\pi -\pi^{\prime }∥}_{1}$. If α=1 then the proposition follows from the rough estimate (9), so from now on, we assume that α<1. Define three probability distributions ${\hat{\pi }}_{0}$, $\pi_{0}^{+}$ and $\pi_{0}^{\prime +}$ on $S_{0}$ by setting for every $x\in S_{0}$

$$\begin{array}{cc} {\hat{\pi }}_{0}\left(x\right)\; & :=\frac{1}{1-\alpha }\min\left\{\pi_{0}\left(x\right),\pi_{0}^{\prime }\left(x\right)\right\}\\ \pi_{0}^{+}\; & :=\frac{1}{\alpha }\left(\pi_{0}-\left(1-\alpha \right){\hat{\pi }}_{0}\right)\\ \pi_{0}^{\prime +}\; & :=\frac{1}{\alpha }\left(\pi_{0}^{\prime }-\left(1-\alpha \right){\hat{\pi }}_{0}\right) \end{array}$$

Denote by $\hat{\pi },\pi^{+},\pi^{\prime +}\in \Delta S$ the distributions corresponding to ${\hat{\pi }}_{0},\pi_{0}^{+},\pi_{0}^{\prime +}\in \Delta S_{0}$ under isomorphism (8). Thus, we have

$$\begin{array}{cc} \pi & =\left(1-\alpha \right)\hat{\pi }+\alpha \cdot \pi^{+}\\ \pi^{\prime } & =\left(1-\alpha \right)\hat{\pi }+\alpha \cdot \pi^{\prime +} \end{array}$$

Now, we construct two fans of G-diagrams
(10)
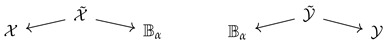

by setting

$$\begin{array}{cc} {\tilde{X}}_{i}\; & :=\left(S_{i}\times \underline{B\;}{\;}_{\alpha };\;{\tilde{\pi }}_{i}\left(s,□\right)=\left(1-\alpha \right){\hat{\pi }}_{i}\left(s\right),\;{\tilde{\pi }}_{i}\left(s,■\right)=\alpha \cdot \pi_{i}^{+}\left(s\right)\right)\\ {\tilde{Y}}_{i}\; & :=\left(S_{i}\times \underline{B\;}{\;}_{\alpha };\;{\tilde{\pi }}_{i}^{\prime }\left(s,□\right)=\left(1-\alpha \right){\hat{\pi }}_{i}\left(s\right),\;{\tilde{\pi }}_{i}^{\prime }\left(s,■\right)=\alpha \cdot \pi_{i}^{\prime +}\left(s\right)\right) \end{array}$$

and

$$\begin{array}{cc} \tilde{X}\; & :=\left\{{\tilde{X}}_{i};\;\sigma_{ij}\times \mathrm{id}\right\}\\ \tilde{Y}\; & :=\left\{{\tilde{Y}}_{i};\;\sigma_{ij}\times \mathrm{id}\right\} \end{array}$$

The reduction in the fans in (10) is given by coordinate projections. Note that the following isomorphisms hold

$$\begin{array}{cc} X|□\; & ≅(S,\hat{\pi })\\ X|■\; & ≅(S,\pi^{+})\\ Y|□\; & ≅(S,\hat{\pi })≅X|□\\ Y|■\; & ≅(S,\pi^{\prime +}) \end{array}$$

Now we apply Proposition 1 along with the rough estimate in (9) to obtain the desired inequality

$$\begin{array}{cc} k(X,Y)\; & \leq (1-\alpha )k(X|□,Y|□)+\alpha \cdot k(X|■,Y|■)\\ \; & \;+\sum_{i}\left[\mathrm{Ent}\left(B_{\alpha }|X_{i}\right)+\mathrm{Ent}\left(B_{\alpha }|Y_{i}\right)\right]\\ \; & \leq 2\left[\;\left[G\right]\;\right]\left(\alpha \cdot \ln|S_{0}|+\mathrm{Ent}\left(B_{\alpha }\right)\right) \end{array}$$

  □

## 5. Proof of Proposition 2

In this section, we prove Proposition 2, which is shown below verbatim. The proof consists of the construction in Section 5.1 and estimates in Propositions 5 and 6.

### 5.1. The Construction

In this section, we fix an indexing category G, a minimal G-diagram of probability spaces Z with an admissible sub-fan $X_{0}\overset{}{\leftarrow }Z_{0}\overset{}{\to }U$. We denote $X:=\left\lceil X_{0}\right\rceil$ and by H we denote the combinatorial type of $X=\left\{X_{i};\chi_{ij}\right\}$.

Instead of diagram Z, we consider an extended diagram, which is a two-fan of H-diagrams
(11)
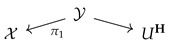

where $Y=\left\{Y_{i};\upsilon_{ij}\right\}$ consists of those spaces in Z, which are initial spaces of two fans with feet in *U* and in some space in X. That is for every i∈H the space $Y_{i}$ is defined to be the initial space in the minimal fan $X_{i}\overset{}{\leftarrow }Y_{i}\overset{}{\to }U$ in Z. It may happen that for some pair of indices $i_{1},i_{2}\in H$ the initial spaces of the fans with one feet *U* and the other $X_{i_{1}}$ and $X_{i_{2}}$ coincide in Z. In Y, however, they will be treated as separate spaces so that the combinatorial type of Y is H. Starting with the diagram in (11) one can recover Z by collapsing all the isomorphism arrows. The initial space of Y will be denoted $Y_{0}$.

We would like to construct a new fan $X^{\prime }\overset{\pi_{1}^{\prime }}{\leftarrow }Y^{\prime }\overset{}{\to }V^{H}$, such that

(12) $$\left\{\begin{array}{cc} X|u=X^{\prime }|v & \mathrm{for}\;\mathrm{any}\;u\in U\;\mathrm{and}\;v\in V\\ k\left(X^{\prime },X\right)\leq 20\left[\;\left[G\right]\;\right]\\ {}[Y_{0}^{\prime }|X_{0}^{\prime }]\leq 4\ln\ln|X_{0}| \end{array}\right.$$

Once this goal is achieved, we collapse all the isomorphisms to obtain G-diagram satisfying conditions in the conclusion of Proposition 2.

We start with a general description of the idea behind the construction, followed by a detailed argument. To introduce the new space *V* we take its points to be *N* atoms in $u_{1},\ldots ,u_{N}\in U$. Ideally, we would like to choose the atoms in such a way that $X_{0}|u_{n}$ are disjoint and cover the whole of $X_{0}$. It is not always possible to achieve this exactly. However, when $|X_{0}|$ is large, *N* is taken slightly larger than $e^{\left[X_{0}:U\right]}$, and $u_{1},\ldots ,u_{N}$ are chosen at random, then with high probability the spaces $X_{0}|u_{n}$ will overlap only little and will cover most of $X_{0}$. The details of the construction follow.

We fix N∈N and construct several new diagrams. For each of the new diagrams, we provide a verbal and formal description.

- The space $U^{N}$. Points in it are independent samples of length *N* of points in *U*.
- The space $V_{N}=\left(\left\{1,\ldots ,N\right\},\mathrm{unif}\right)$. A point $n\in V_{N}$ should be interpreted as a choice of index in a sample $\bar{u}\in U^{N}$.
- The H-diagram A, where
  $$\begin{array}{cc} A\; & =\left\{A_{i};\;\alpha_{ij}\right\}\\ A_{i}\; & =\left(\left\{\left(x,n,\bar{u}\right)\;:\;x\in X_{i}|u_{n}\right\},\mathrm{unif}\right)\\ \alpha_{ij}\; & =(\chi_{ij},\mathrm{Id},\mathrm{Id}) \end{array}$$
  A point $\left(x,n,\bar{u}\right)$ in $A_{i}$ corresponds to the choice of a sample $\bar{u}\in U^{N}$, an independent choice of a member of the sample $u_{n}$ and a point $x\in X_{i}|u_{n}$. Recall that the original diagram Z was assumed to be homogeneous and, in particular, the distribution on $X_{i}|u_{n}$ is uniform. Due to the assumption on homogeneity of Z, the space $X_{i}|u$ does not depend on u∈U. Since $V_{N}$ is also equipped with the uniform distribution, it follows that the distribution on $A_{i}$ will also be uniform.
- The H-diagram B, where
  $$\begin{array}{cc} B\; & =\left\{B_{i};\;\beta_{ij}\right\}\\ B_{i}\; & =\left(\left\{\left(x,\bar{u}\right)\;:\;x\in \overset{N}{\underset{n=1}{⋃}}X_{i}|u_{n}\right\},p_{B_{i}}\right)\\ \beta_{ij}\; & =(\chi_{ij},\mathrm{Id}) \end{array}$$
  A point $\left(x,\bar{u}\right)\in B_{i}$ is the choice of a sample $\bar{u}\in U^{N}$ and a point *x* in one of the fibers $X_{i}|u_{n}$, n=1,…,N. The distribution $p_{B_{i}}$ on $B_{i}$ is chosen so that the natural projection $A_{i}\overset{}{\to }B_{i}$ is the reduction of probability spaces. Given a sample $\bar{u}$, if the fibers $X_{i}|u_{n}$ are not disjoint, then the distribution on $B_{i}|\bar{u}$ need not to be uniform. Below, we will give an explicit description of $p_{B}$ and study the dependence of $p_{B}\left(\;\cdot \;|\bar{u}\right)$ on the sample $\bar{u}\in U^{N}$.

These diagrams can be organized into a minimal diamond diagram of H-diagrams, where reductions are obvious projections.
(13)
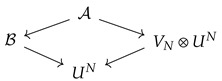


To describe the probability distribution on B, first we define several relevant quantities:

(14) $$\begin{array}{cc} \rho \; & :=\frac{|X_{0}\left|u\right|}{|X_{0}|}=e^{-[X_{0}:U]}\\ N(x,\bar{u})\; & :=|\left\{n\in V_{N}\;:\;x\in X_{0}|u_{n}\right\}|\\ \nu (x,\bar{u})\; & :=\frac{N(x,\bar{u})}{N}=p_{V_{N}}\left\{n\in V_{N}\;:\;x\in X_{0}|u_{n}\right\} \end{array}$$

Recall that the distribution $p_{B}$ is completely determined by the distribution $p_{B_{0}}$ on the initial space of B via isomorphism (8). From homogeneity of Z it follows that distributions on both $A_{0}$ and ${A|}_{\bar{u}}$ are uniform. Therefore

(15) $$p_{B_{0}}\left(x|\bar{u}\right):=\frac{\nu (x,\bar{u})}{\rho \cdot |X_{0}|}$$

The desired fan $\left(X^{\prime }\overset{}{\leftarrow }Y^{\prime }\overset{}{\to }V^{H}\right)$ mentioned in the beginning of the section is obtained from the top fan in the diagram in (13) by conditioning on $\bar{u}\in U^{N}$. We will show later that for an appropriate choice of *N* and for most choices of $\bar{u}$, the fan we obtain in this way has the required properties.

First, we would like to make the following observations. Fix an arbitrary $\bar{u}\in U^{N}$. Then:

(1) The underlying set of the probability space $B_{0}\left|\bar{u}=X_{0}\right|\bar{u}$ is $\underline{X\;}{\;}_{0}$.
(2) The diagrams
  $$\begin{array}{cc} Y_{\bar{u}}^{\prime }\; & :=A|\bar{u}\\ X_{\bar{u}}^{\prime }\; & :=B|\bar{u} \end{array}$$
  are included in a two-fan of H-diagrams

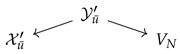

which is obtained by conditioning the top fan in the diagram in (13).
  The very important observation is that diagrams $X_{\bar{u}}^{\prime }|n$ and X|u are isomorphic for any choice of $n\in V_{N}$ and u∈U. The isomorphism is the composition of the following sequence of isomorphisms
  $$X_{\bar{u}}^{\prime }|n\overset{}{\to }B|\left(\bar{u},n\right)\overset{}{\to }A|\left(\bar{u},n\right)\overset{}{\to }X\left|u_{n}\overset{}{\to }X\right|u$$
  where the first isomorphism follows from the definition of $X_{\bar{u}}^{\prime }$, the second—from minimality of the fan $B\overset{}{\leftarrow }A\overset{}{\to }V_{N}$, the third—from the definition of A and the fourth—from the homogeneity of Z.

### 5.2. The Estimates

We now claim and prove that one could choose a number *N* and $\bar{u}$ in $U^{N}$ such that

(1) $k\left(X_{\bar{u}}^{\prime },X\right)\leq 20\left[\;\left[H\right]\;\right]$.
(2) $\left[Y_{\bar{u},0}^{\prime }|X_{\bar{u},0}^{\prime }\right]\leq 4\ln\ln\left|X_{0}\right|$, where $Y_{\bar{u},0}^{\prime }$ and $X_{\bar{u},0}^{\prime }$ are initial spaces in $X_{\bar{u}}^{\prime }$ and $Y_{\bar{u}}^{\prime }$, respectively.

#### 5.2.1. Total Variation and Entropic Distance Estimates

If we fix some $x_{0}\in X_{0}$, then $\nu =\nu (x_{0},\;\cdot \;)$ is a scaled binomially distributed random variable with parameters *N* and ρ, which means that N·ν∼Bin(N,ρ).

First, we state the following bounds on the tails of a binomial distribution.

**Lemma 1.**  *Let ν be a scaled binomial random variable with parameters N and ρ, then*

*(i)*  *for any t∈[0,1] holds*
$$P\left\{|\nu -\rho |>\rho \cdot t\right\}\leq 2\cdot e^{-\frac{1}{3}\cdot N\cdot \rho \cdot t^{2}}$$
*(ii)*  *for any t∈[0,2] holds*
$$P\left\{\frac{\nu }{\rho }\ln\frac{\nu }{\rho }>t\right\}\leq e^{-\frac{1}{12}\cdot N\cdot \rho \cdot t^{2}}$$

The proof of Lemma 1 can be found at the end of this section.

Below we use the notation $P:=p_{U^{N}}$ for the probability distribution on $U^{N}$. For a pair of complete diagrams C, $C^{\prime }$ with the same underlying diagram of sets and with initial spaces $C_{0}$, $C_{0}^{\prime }$, we will write $\alpha (C,C^{\prime })$ for the halved total variation distance between their distributions

$$\alpha \left(C,C^{\prime }\right):=\frac{1}{2}{∥p_{C_{0}}-p_{C_{0}^{\prime }}∥}_{1}$$

**Proposition 4.**  *In the settings above, for t∈[0,1], the following inequality holds*

$$P\left\{\bar{u}\in U^{N}\;:\;2\alpha \left(X_{\bar{u}}^{\prime },X\right)>t\right\}\leq 2\left|X_{0}\right|\cdot e^{-\frac{1}{3}N\cdot \rho \cdot t^{2}}$$

**Proof.**  Recall that by definition $X_{\bar{u}}^{\prime }=B|\bar{u}$. We use Equation (15) to expand the left-hand side of the inequality as follows

$$\begin{array}{l} P\left\{\bar{u}\in U^{N}\;:\;2\alpha \left(B|\bar{u},X\right)>t\right\}=P\left\{\bar{u}\in U^{N}\;:\;\sum_{x\in X_{0}}\left|\frac{\nu (x,\bar{u})}{\rho \cdot |X_{0}|}-\frac{1}{|X_{0}|}\right|>t\right\}\\ \;=P\left\{\bar{u}\in U^{N}\;:\;\sum_{x\in X_{0}}\left|\nu (x,\bar{u})-\rho \right|>\rho \cdot \left|X_{0}\right|\cdot t\right\}\\ \;\leq P\left\{\bar{u}\in U^{N}\;:\;\mathrm{there}\;\mathrm{exists}\;x_{0}\;\mathrm{such}\;\mathrm{that}\;\left|\nu (x_{0},\bar{u})-\rho \right|>\rho \cdot t\right\}\\ \;\leq \sum_{x\in X_{0}}P\left\{\bar{u}\in U^{N}\;:\;\left|\nu (x,\bar{u})-\rho \right|>\rho \cdot t\right\} \end{array}$$

Since by homogeneity of the original diagram, all the summands are the same, we can fix some $x_{0}\in X_{0}$ and estimate further:

$$\begin{array}{c} P\left\{\bar{u}\in U^{N}\;:\;2\alpha \left(B|\bar{u},X\right)>t\right\}\leq \left|X_{0}\right|\cdot P\left\{\bar{u}\in U^{N}\;:\;\left|\nu (x_{0},\bar{u})-\rho \right|>\rho \cdot t\right\} \end{array}$$

Applying Lemma 1(i), we obtain the required inequality. □

In the propositions below we assume that $|X_{0}|$ is sufficiently large (larger than $e^{20}$).

**Proposition 5.**  *In the settings above and for any $\frac{10}{\ln|X_{0}|}\leq t\leq 1$ holds:*

$$P\left\{\bar{u}\in U^{N}:k\left(X_{\bar{u}}^{\prime },X\right)>t(2\cdot \left[\;\left[G\right]\;\right]\cdot \ln|X_{0}\left|\right)\right\}\leq 2\left|X_{0}\right|\cdot e^{-\frac{1}{3}N\cdot \rho \cdot t^{2}}$$

**Proof.**  We will use local estimate, Proposition 3, to bound the entropy distance and then apply Proposition 4. To simplify notations, we will write simply α for $\alpha \left(X_{\bar{u}}^{\prime },X\right)=\alpha \left(B|\bar{u},X\right)$.

$$\begin{array}{c} P\left\{\bar{u}\in U^{N}:k\left(B|\bar{u},X\right)>(2\cdot \left[\;\left[G\right]\;\right]\cdot \ln|X_{0}\left|\right)t\right\}\\ \;\leq P\left\{\bar{u}\in U^{N}:2\cdot \left[\;\left[G\right]\;\right](\alpha \cdot \ln|X_{0}|+\mathrm{Ent}\left(\Lambda_{\alpha }\right))>(2\cdot \left[\;\left[G\right]\;\right]\cdot \ln|X_{0}\left|\right)t\right\}\\ \;\leq P\left\{\bar{u}\in U^{N}:\alpha +\mathrm{Ent}\left(\Lambda_{\alpha }\right)/\ln\left|X_{0}\right|>t\right\} \end{array}$$

Please note that in the chosen regime, $t\geq 10/\ln|X_{0}|$, the first summand in the right-hand side of the inequality is larger than the second, i.e., $\alpha \geq \mathrm{Ent}\left(\Lambda_{\alpha }\right)/\ln\left|X_{0}\right|$ and therefore we can write

$$\begin{array}{c} P\left\{\bar{u}\in U^{N}:k\left(B|\bar{u},X\right)>(2\cdot \left[\;\left[G\right]\;\right]\cdot \ln|X_{0}\left|\right)t\right\}\\ \;\leq P\left\{\bar{u}\in U^{N}:2\alpha >t\right\}\\ \;\leq 2|X_{0}|\cdot e^{-\frac{1}{3}N\cdot \rho \cdot t^{2}} \end{array}$$

  □

#### 5.2.2. The “Height” Estimate

Recall that for given N∈N and $\bar{u}\in U^{N}$ we have constructed a two-fan of H-diagrams

$$X_{\bar{u}}^{\prime }\overset{}{\leftarrow }Y_{\bar{u}}^{\prime }\overset{}{\to }V_{N}^{H}$$

We will now estimate the length of the arrow $Y_{\bar{u},0}^{\prime }\overset{}{\to }X_{\bar{u},0}^{\prime }$.

**Proposition 6.**  *In the settings above and for t∈[0,2]*

$$P\left\{\bar{u}\in U^{N}\;:\;\left[Y_{\bar{u},0}^{\prime }|X_{\bar{u},0}^{\prime }\right]>\ln\left(N\cdot \rho \right)+t\right\}\leq \left|X_{0}\right|\cdot e^{-\frac{1}{12}N\cdot \rho \cdot t^{2}}$$

**Proof.**  First, we observe that the fiber of the reduction $Y_{\bar{u},0}^{\prime }\overset{}{\to }X_{\bar{u},0}^{\prime }$ over a point $x\in X_{\bar{u},0}^{\prime }$ is a homogeneous probability space of cardinality equal to $N(x,\bar{u})$, therefore its entropy is $\ln N(x,\bar{u})$.

$$\begin{array}{c} P\left\{\bar{u}\in U^{N}\;:\;\left[Y_{\bar{u},0}^{\prime }|X_{\bar{u},0}^{\prime }\right]>\ln\left(N\cdot \rho \right)+t\right\}\\ P\left\{\bar{u}\in U^{N}\;:\;\int_{X_{\bar{u},0}^{\prime }}\left[Y_{\bar{u},0}^{\prime }|x\right]\;dp_{X_{\bar{u},0}^{\prime }}\left(x\right)>\ln\left(N\cdot \rho \right)+t\right\}\\ \;=P\left\{\bar{u}\in U^{N}\;:\;\sum_{x\in X_{0}}\frac{\nu (x,\bar{u})}{\rho \cdot |X_{0}|}\ln\left(N\cdot \nu (x,\bar{u})\right)>\ln\left(N\cdot \rho \right)+t\right\}\\ \;\leq P\left\{\bar{u}\in U^{N}\;:\;\sum_{x\in X_{0}}\frac{\nu (x,\bar{u})}{\rho \cdot |X_{0}|}\ln\left(\frac{\nu (x,\bar{u})}{\rho }\right)>t\right\}\\ \;\leq |X_{0}|\cdot P\left\{\bar{u}\in U^{N}\;:\;\frac{\nu (x_{0},\bar{u})}{\rho }\ln\left(\frac{\nu (x_{0},\bar{u})}{\rho }\right)>t\right\}\\ \;\leq |X_{0}|\cdot e^{-\frac{1}{12}N\cdot \rho \cdot t^{2}} \end{array}$$

The last inequality above follows from Lemma 1 (ii). □

### 5.3. Proof of Proposition 2

Let $X_{\bar{u}}^{\prime }\overset{}{\leftarrow }Y_{\bar{u}}^{\prime }\overset{}{\to }V_{N}$ be the fan constructed in Section 5.1. The construction is parameterized by number *N* and atom $\bar{u}\in U^{N}$. Below, we will choose a particular value for *N* and apply estimates in Propositions 5 and 6 with particular choice of parameter *t* to show that there is $\bar{u}\in U^{N}$, so that the fan satisfies the conclusions of Proposition 2.

Let

$$\begin{array}{l} N\;:=\ln^{3}|X_{0}|\cdot \rho^{-1}=\ln^{3}\left|X_{0}\right|\cdot e^{\left[X_{0}:U\right]}\\ \;t\;:=\frac{10}{\ln|X_{0}|} \end{array}$$

With these choices of *N* and *t*, Proposition 5 implies

$$P\left\{\bar{u}\in U^{N}\;:\;k\left(X_{\bar{u}}^{\prime },X\right)>20\left[\;\left[G\right]\;\right]\right\}\leq \frac{1}{4}$$

while Proposition 6 gives

$$P\left\{\bar{u}\in U^{N}\;:\;\left[Y_{\bar{u},0}^{\prime }|X_{\bar{u},0}^{\prime }\right]>4\ln\ln\left|X_{0}\right|\right\}\leq \frac{1}{4}$$

Therefore, there is a choice of $\bar{u}$ such that the fan

$$\left(X^{\prime }\overset{}{\leftarrow }Y^{\prime }\overset{}{\to }V\right):=\left(X_{\bar{u},0}^{\prime }\overset{}{\leftarrow }Y_{\bar{u},0}^{\prime }\overset{}{\to }V_{N}\right)$$

satisfies conditions in (12). As we have explained at the beginning of Section 5.1, by collapsing isomorphism arrows, we obtain G-diagram $Z^{\prime }$ satisfying conclusions of Proposition 2.

### 5.4. Proof of Lemma 1

The Chernoff bound for the tail of a binomially distributed random variable X∼Bin(N,ρ) asserts that for any 0≤δ≤1 holds

$$\begin{array}{c} P\left\{X<(1-\delta )N\cdot \rho \right\}\;\leq e^{-\frac{1}{2}\delta^{2}N\cdot \rho }\\ P\left\{X>(1+\delta )N\cdot \rho \right\}\;\leq e^{-\frac{1}{3}\delta^{2}N\cdot \rho } \end{array}$$

Applying the bound for the upper and lower tail for the binomially distributed random variable N·ν, we obtain the inequality in (i).

The second assertion follows from the following estimate

$$\begin{array}{cc} P\left\{\frac{\nu }{\rho }\ln\frac{\nu }{\rho }>t\right\}\; & \leq P\left\{\frac{\nu }{\rho }\left(\frac{\nu }{\rho }-1\right)>t\right\}\\ \; & =P\left\{\nu >\rho \cdot \left(\frac{\sqrt{1+4t}-1}{2}+1\right)\right\} \end{array}$$

For 0≤t≤2 we have $\sqrt{1+4t}-1\geq t$, therefore

$$P\left\{\frac{\nu }{\rho }\ln\frac{\nu }{\rho }>t\right\}\;\leq P\left\{\nu >\rho \cdot \left(\frac{t}{2}+1\right)\right\}$$

By the Chernoff bound, we have

$$P\left\{\frac{\nu }{\rho }\ln\frac{\nu }{\rho }>t\right\}\;\leq e^{-\frac{1}{12}N\cdot \rho \cdot t^{2}}$$

## Data Availability

No new data were created or analyzed in this study. Data sharing is not applicable to this article.
